# Associations between White Blood Cell Count and the Development of Incidental Nonalcoholic Fatty Liver Disease

**DOI:** 10.1155/2016/7653689

**Published:** 2016-12-13

**Authors:** Goh Eun Chung, Jeong Yoon Yim, Donghee Kim, Min-Sun Kwak, Jong In Yang, Su Jin Chung, Sun Young Yang, Joo Sung Kim

**Affiliations:** ^1^Department of Internal Medicine, Healthcare Research Institute, Gangnam Healthcare Center, Seoul National University Hospital, Seoul, Republic of Korea; ^2^Division of Gastroenterology and Hepatology, Stanford University School of Medicine, Stanford, CA, USA; ^3^Department of Internal Medicine and Liver Research Institute, Seoul National University College of Medicine, Seoul, Republic of Korea

## Abstract

*Aims*. Chronic low-grade inflammation is thought to be associated with the pathogenesis of nonalcoholic fatty liver disease (NAFLD). This study aimed to determine the association between serum white blood cell (WBC) counts and the development of incidental NAFLD.* Methods*. In this retrospective longitudinal cohort study, we recruited participants who underwent abdominal ultrasonography and blood samplings during medical checkups in both 2005 and 2010. A total of 2,216 subjects were included in our analyses.* Results*. The prevalence of NAFLD in 2010 increased steadily in conjunction with increasing WBC counts in 2005 after adjustment for body mass index (BMI) [odds ratio (OR) 2.44, 95% confidence interval (CI) = 1.49–4.00 for women and OR 2.42, 95% CI = 1.61–3.63 for men, lowest quartile versus highest quartile]. Multivariate regression analysis after adjusting for age, BMI, hypertension, smoking, triglycerides, HDL cholesterol, and glucose levels revealed that NAFLD was significantly associated with the highest WBC quartile compared to the lowest quartile [OR 1.85, 95% CI, 1.10−3.10 for women and OR 1.68, 95% CI, 1.08−2.61 for men].* Conclusions*. We demonstrated that the risk of developing NAFLD was significantly associated with WBC counts independently of metabolic factors. This finding provides novel evidence indicating that serum WBC counts may be potential surrogate markers of NAFLD.

## 1. Introduction

White blood cell (WBC) counts are routinely measured in clinical practice as markers of systemic inflammation. Elevated WBC counts have been associated with various diseases such as cardiovascular disease, infection, diabetes, metabolic syndrome (MS) and nonalcoholic fatty liver disease (NAFLD) [1–4], conditions related to insulin resistance, and chronic low-grade inflammation [5, 6].

NAFLD is the most common chronic liver disease and its prevalence has increased to 20–30% in Western nations and to 16–33% in Korea [7, 8], and it leads to steatohepatitis, fibrosis, cirrhosis, and hepatocellular carcinoma in some individuals [9]. NAFLD is closely associated with risk factors for cardiovascular diseases, including central obesity, dyslipidemia, hypertension, and glucose intolerance, all of which are components of metabolic syndrome [10]. Given that the effects of NAFLD were not limited to the liver, it is very important to predict NAFLD and to modify its risk factors.

To date, few studies have examined the longitudinal relationship between serum WBC counts and the development of incidental MS and NAFLD. The purpose of the current study was to investigate the causal relationship between serum WBC count and the development of incidental NAFLD, adjusting for metabolic risk factors in a Korean population.

## 2. Patients and Methods

### 2.1. Study Population

We analysed data from a previously described longitudinal cohort study [11]. Briefly, subjects who underwent abdominal ultrasonography (US) and blood samplings at the Seoul National University Hospital Gangnam Healthcare Center, Seoul, Korea, during routine health checkups in both 2005 and 2010 were initially included in this cohort. We excluded the following subjects because they had a potential cause of chronic liver disease: 754 subjects who had a history of significant alcohol intake (>20 g/day for males and >10 g/day for females) [12], 184 subjects who were positive for hepatitis B virus infection, and 44 subjects who were positive for hepatitis C virus infection. Among the 3,473 subjects who were initially enrolled in this study, 1,168 (33.6%) were excluded because of preexisting NAFLD and 89 were excluded because of missing data. In addition, 26 subjects with WBC count ≥10,000 cells/*μ*L were excluded to rule out other related conditions such as infection or chronic inflammation. This study was approved by the Institutional Review Board of the Seoul National University Hospital. The informed consent requirement was waved.

### 2.2. Clinical and Laboratory Assessments

Each subject completed a routine previous medical history questionnaire and underwent an anthropometric assessment in addition to laboratory and radiological tests on the same day. Body weight and height were measured using a digital scale. Body mass index (BMI) was calculated as the ratio of weight (kg)/height squared (m^2^). Waist circumference at the midpoint between the lower costal margin and the anterior superior iliac crest was measured by a well-trained person. Systolic and diastolic blood pressures were measured twice and their mean values were reported. After a 12-hour fast, blood samples were collected from the antecubital vein of each individual. WBC counts were quantified using an automated blood cell counter (ADVIA 2120, Bayer, NY, USA). Laboratory evaluations of the patients also included measurements of serum alanine aminotransferase, total cholesterol, triglyceride, high-density lipoprotein (HDL) cholesterol, and fasting glucose levels, hepatitis B surface antigen, and antibody to hepatitis C virus. Diabetes mellitus was defined as either a fasting serum glucose level ≥126 mg/dL or a history of antidiabetic medication use. Hypertension was defined as having a systolic blood pressure ≥140 mmHg or a diastolic blood pressure ≥90 mmHg or a history of antihypertensive medication use. For smoking status, patients were categorized as nonsmokers, former smokers, or current smokers [13].

### 2.3. Ultrasonographic Examinations

NAFLD was diagnosed based on the findings of ultrasonography (Acusion, Sequoia 512, Siemens, Mountain View, CA) by experienced radiologists These radiologists were unaware of any relevant clinical information. Fatty liver was characterized by the followings ultrasonographic features: bright echoes in the liver, high hepatorenal echogenicity, or deep attenuation and impaired visualization of the diaphragm and marked vascular blurring [14]. Follow-up hepatic ultrasonography was conducted according to the same protocol and using the same equipment as used at baseline.

### 2.4. Statistical Analysis

Patients stratified into quartiles according to WBC count were categorized separately by gender because WBC counts differ significantly between sexes. The following ranges were applied: Q1: ≤4300, Q2: 4310−5040, Q3: 5050–6030, and Q4: ≥6040 cells/*μ*L for women and Q1: ≤4710, Q2: 4720–5500, Q3: 5510–64550, and Q4: ≥6460 cells/*μ*L for men. Analysis of variance (ANOVA) and the linear-by-linear association test were used to compare the characteristics of study subjects according to WBC count quartiles. Variables that were statistically significant based on univariate analysis and known risk factors were added to a multiple logistic regression model to identify the independent predictors of NAFLD development. The area under receiver operating characteristic curve (AUROC) analysis was used to determine effective cutoff values of WBC counts for detecting subjects with NAFLD. To obtain the optimal cutoff point to discriminate the disease from the nondisease subject, we calculated the distance from the left-upper corner of the unit square to any point on the RUC curve [distance = (1 − sensitivity)^2^ + (1 − specificity)^2^] for each observed cutoff point and chose the point where the distance is minimum [15]. All statistical analyses were performed using SPSS 22.0 (SPSS Inc.; Chicago, IL, USA). *P* values < 0.05 were considered statistically significant.

## 3. Results

A total of 2,190 subjects were included in the analysis. The mean age of these participants was 48.1 ± 9.6 years old and 43.1% were male.  Table 1 presents the baseline characteristics of the study population across the quartiles stratified according to serum WBC count. The mean BMI, triglyceride, and fasting glucose levels were highest in the 4th quartiles of WBC count whereas HDL cholesterol was lowest in the 4th quartile of WBC count in both female and male patients. The rates of NAFLD development were significantly higher in the 4th quartile of both male and female patients stratified according to WBC count than in the other three quartiles.

We analysed the relationship between the risk of developing NAFLD in 2010 and the serum WBC count in 2005. A total of 565 (25.5%) subjects developed incidental NAFLD during the follow-up period. To analyse the relationship between NAFLD and WBC count_,_ multivariable binary and ordinal analyses were performed with NAFLD serving as a dependent variable (Table 2). The prevalence of NAFLD increased steadily with increasing WBC counts after adjustment for age and BMI [odds ratio (OR) 2.44, 95% confidence interval (CI) = 1.49–4.00 for women and OR 2.42, 95% CI = 1.61–3.63 for men, lowest quartile versus highest quartile]. Multivariate regression analysis after adjusting for age, BMI, hypertension, smoking, triglycerides, HDL cholesterol, and glucose levels revealed that NAFLD was significantly associated with the highest WBC quartile compared to the lowest quartile [OR 1.85, 95% CI, 1.10−3.10 for women and OR 1.68, 95% CI, 1.08−2.61 for men].

**Figure 1:**
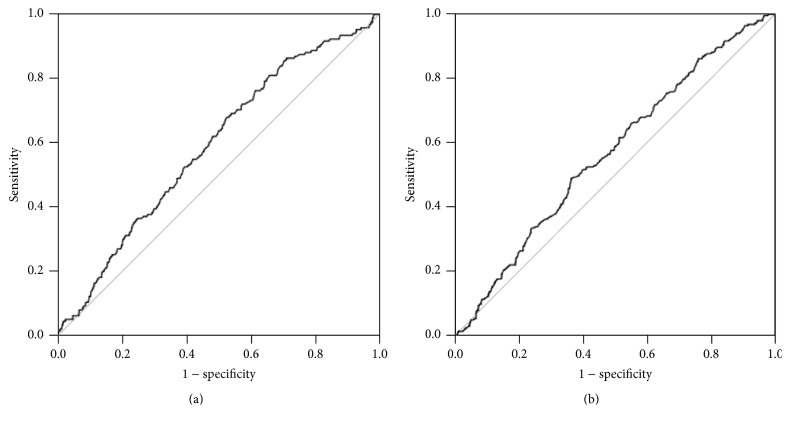
Receiver operating characteristic (ROC) curve of white blood cell (WBC) counts to detect nonalcoholic fatty liver disease (NAFLD) in women (a) and men (b).

The AUROC was 0.566 (95% CI, 0.526–0.607) for men and 0.590 (95% CI, 0.545–0.634) for women (Figure 1). The sensitivity and specificity for NAFLD in women were 56.0% and 58.5%, respectively, using a cutoff WBC count of 5,226 cells/*μ*L. The sensitivity and specificity for NAFLD in men were 53.7% and 64.4%, respectively, using a cutoff WBC count of 5,767 cells/*μ*L.

## 4. Discussion

In this study, serum WBC counts were associated with the risk of developing incidental NAFLD, and this association was independent of metabolic risk factors. Previous studies have demonstrated an association between WBC counts and NAFLD [4, 16]. However, these studies have cross-sectional designs; thus it was impossible to draw strong conclusions regarding the existence of causal relationships between WBC counts and NAFLD or the temporal sequence of events linking the two variables. In this study, we demonstrated that higher WBC counts were associated with greater risk of developing incidental NAFLD during the 5-year follow-up period after adjustment for other confounding factors. This finding provides novel evidence indicating that the serum WBC count may be a surrogate marker of NAFLD.

Consistent with our research, a recent study performed in China has also investigated the value of the WBC count as an independent predictor of the risk of developing NAFLD [17]. That study determined that compared with the lowest quartile of patients stratified according to WBC count, the other WBC quartiles were significantly associated with increased risk of NAFLD after adjustment for other relevant factors. However, although gender was accounted for during the analysis, the results of that study were not stratified according to gender and the criteria used to determine quartiles of patients based on WBC count were not gender-specific. In contrast, we analysed the data according to gender as WBC counts differ significantly between men and women [18, 19].

The mechanisms linking elevated serum WBC counts to NAFLD development remain to be determined. Several possible explanations are available. (i) Insulin resistance is the critical step in the development of NAFLD and serves as a source of oxidative stress, thereby, activating inflammatory processes [20]. Hyperinsulinemia causes increase in hepatic de novo lipogenesis and adipose tissue lipolysis, leading to greater transport of free fatty acids to the liver and the development of steatosis, which is potentiated by multiple pathogenic and injurious factors. Activation of the transforming growth factor-beta pathway, dysregulation of multiple adipokines, apoptosis or activation of liver stellate cells and oxidative stress may induce hepatic inflammation [21]. (ii) Obesity is associated with chronic systemic low-grade inflammation in which adipose tissue plays a central role. Adipose tissue is an active endocrine organ with the capacity to synthesize and secrete various adipokines and cytokines in both healthy and disease states. Adipose tissue imbalances caused by obesity induce ectopic fat accumulation in the liver, leading to NAFLD; furthermore, these imbalances increase secretion of proinflammatory cytokines such as tumor necrosis factor (TNF) and interleukin- (IL-) 6 and reduce the secretion of anti-inflammatory factors such as IL-10 and adiponectin [22]. Many studies have demonstrated significant associations between NAFLD and inflammatory molecules and cytokines, suggesting the important role of inflammation in the pathogenesis of NAFLD [23, 24]. (iii) Because the liver, connected by the portal system, is the site of direct venous outflow from intestine, the liver is vulnerable to microbiome translocation, microbial products, and endotoxins. The microbiome is affected by diet, bile acid levels, and other factors and is related to NAFLD. Alterations to the microbiome may stimulate liver steatosis by increasing intestinal permeability and low-grade inflammation as a result of exposing the liver to high concentrations of harmful intestinal contents [25, 26]. Certain microbiome members, such as Escherichia, produce endogenous alcohol resulting in reactive oxygen species production and liver inflammation [27]. In addition, microbial products, such as lipopolysaccharides, are potentially cytotoxic and induce inflammation. Lipopolysaccharides are recognized by Toll-like receptors which are known to be associated with liver injury through a mechanism in which stimulation of Toll-like receptors activates several signaling cascades, especially the NF-*κ*B, pathway to induce the expression of inflammatory cytokines and chemokines [28–30]. Low-grade metabolic endotoxemia was reported to be associated with NAFLD [31, 32]. Collectively, these explanations indicate that hepatic inflammation may precede simple steatosis and various hepatic stresses can influence the development and progression of low-grade systemic inflammation, which may lead to NAFLD. These findings are supported by several studies demonstrating that anti-TNF antibody treatment or metformin, which inhibits hepatic TNF-*α* expression, alleviates hepatic steatosis [33–35] and that multiple dysregulation-inducing processes may simultaneously contribute to the development NAFLD and low-grade systemic inflammation.

There are several limitations to this study. First, because the study included subjects who visited a single health screening center in Korea for health checkups, the study population may not be representative of the general population and our results may have been affected by selection bias. Second, only one serum WBC count measurement was included in the analysis. Therefore, we excluded subjects with WBC count ≥10,000 cells/*μ*L to rule out other related conditions such as infection or chronic inflammation. Third, we could not evaluate the direct effects of insulin resistance because we did not obtain data regarding insulin levels. Finally, we obtained no data related to chronic inflammatory markers that may be associated with NAFLD, such as IL-6 or C-reactive protein.

In conclusion, we have demonstrated that serum WBC counts are independently associated with the risk of developing incidental NAFLD during a 5-year follow-up period. This finding serves as novel evidence indicating that the serum WBC count may be a potential surrogate marker of NAFLD.

## Figures and Tables

**Table 1:** Baseline characteristics of study subjects according to white blood cell quartiles.

Women (cells/*μ*L)	Q1 (*N* = 313)	Q2 (*N* = 313)	Q3 (*N* = 313)	Q4 (*N* = 313)	*P* for trend
Age, years	47.0 ± 9.2	48.0 ± 9.2	46.8 ± 9.3	46.6 ± 9.4	0.243
Body mass index, kg/m^2^	21.2 ± 2.2	21.8 ± 2.4	21.9 ± 2.4	22.4 ± 2.9	<0.001
Waist circumference, cm	79.1 ± 6.7	79.8 ± 6.7	79.8 ± 6.9	80.8 ± 7.5	0.017
ALT, IU/L	18.8 ± 14.3	16.7 ± 8.6	17.4 ± 9.4	17.4 ± 10.1	0.090
Fasting glucose, mg/dL	92.4 ± 10.5	93.9 ± 8.8	95.5 ± 14.0	94.3 ± 12.6	0.010
Triglycerides, mg/dL	70.5 ± 29.9	84.1 ± 42.4	85.9 ± 43.2	91.6 ± 50.9	<0.001
HDL cholesterol, mg/dL	62.4 ± 13.8	60.7 ± 13.1	59.5 ± 13.1	56.6 ± 12.6	<0.001
White blood cell counts, cells/*μ*L	37.8 ± 4.3	46.6 ± 2.1	54.8 ± 2.9	70.6 ± 8.9	<0.001
Current smoking, %	6 (1.9)	11 (3.5)	2 (0.6)	11 (3.5)	0.072
Hypertension, %	32 (10.2)	28 (8.9)	24 (7.7)	43 (13.7)	0.071
5 yr_NAFLD, %	29 (9.3)	55 (17.6)	58 (17.9)	78 (24.9)	<0.001

Men (cells/*μ*L)	Q1 (*N* = 238)	Q2 (*N* = 239)	Q3 (*N* = 239)	Q4 (*N* = 239)	*P* for trend

Age, years	48.8 ± 9.3	50.5 ± 10.0	50.1 ± 10.2	49.1 ± 9.4	0.171
Body mass index, kg/m^2^	23.3 ± 2.4	23.8 ± 2.3	24.3 ± 2.3	24.2 ± 2.3	<0.001
Waist circumference, cm	83.6 ± 6.5	85.4 ± 6.3	86.7 ± 6.3	86.5 ± 6.3	<0.001
ALT, IU/L	22.1 ± 13.1	22.3 ± 10.8	24.2 ± 10.0	25.2 ± 13.9	0.012
Fasting glucose, mg/dL	100.1 ± 13.8	102.2 ± 16.3	104.3 ± 17.8	104.7 ± 21.5	0.020
Triglycerides, mg/dL	95.5 ± 47.0	114.8 ± 59.4	125.3 ± 84.1	142.6 ± 74.3	<0.001
HDL cholesterol, mg/dL	53.6 ± 11.8	49.9 ± 11.6	48.9 ± 10.3	46.9 ± 10.2	<0.001
White blood cell counts, cells/*μ*L	41.8 ± 3.9	51.0 ± 2.1	59.4 ± 2.7	75.5 ± 8.5	<0.001
Current smoking, %	34 (14.7)	46 (19.7)	68 (28.8)	103 (44.0)	<0.001
Hypertension, %	32 (13.9)	47 (20.1)	46 (19.5)	51 (21.8)	0.145
5 yr_NAFLD, %	58 (25.1)	77 (32.9)	91 (38.6)	113 (48.3)	<0.001

Values are expressed as the mean ± standard deviation or number (%).

ALT: alanine aminotransferase; NAFLD: nonalcoholic fatty liver disease; HDL: high density lipoprotein.

**Table 2:** Age- and sex-adjusted multivariate analyses of the risk of developing incidental NAFLD at 5 years after the baseline examination according to white blood cell quartiles.

	Q1	Q2	Q3	Q4
Women				
Model 1	1 (ref)	2.09 (1.29–3.38)^*∗*^	2.13 (1.32–3.45)^*∗*^	2.05 (2.05–5.15)^*∗*^
Model 2	1 (ref)	1.76 (1.06–2.92)^*∗*^	1.82 (1.09–3.02)^*∗*^	2.44 (1.49–4.00)^*∗*^
Model 3	1 (ref)	1.51 (0.90–2.55)	1.40 (0.82–2.37)	1.85 (1.10–3.10)^*∗*^
Men				
Model 1	1 (ref)	1.44 (0.97–2.14)	1.96 (1.32–2.89)^*∗*^	2.57 (1.75–3.79)^*∗*^
Model 2	1 (ref)	1.33 (0.88–2.03)	1.54 (1.02–2.32)^*∗*^	2.42 (1.61–3.63)^*∗*^
Model 3	1 (ref)	1.15 (0.75–1.77)	1.23 (0.80–1.89)	1.68 (1.08–2.61)^*∗*^

Mode 1: unadjusted. Model 2: adjusting for age and body mass index. Model 3: adjusting for age, body mass index, hypertension, smoking, glucose, HDL cholesterol, and triglyceride.

^*∗*^ *P* < 0.05.

NAFLD: nonalcoholic fatty liver disease; HDL: high density lipoprotein.

## Abbreviations

BMI: Body mass index
HDL: High-density lipoprotein
NAFLD: Nonalcoholic fatty liver disease
WBC: White blood cell
TNF: Tumor necrosis factor
IL: Interleukin
ROC: Receiver operating characteristic.
